# Cardiac Structural Remodeling and Hemodynamic Patterns Following Transcatheter Aortic Valve Replacement

**DOI:** 10.7759/cureus.19224

**Published:** 2021-11-03

**Authors:** Julien Feghaly, Debapria Das, Zachary Oman, Steven Smart

**Affiliations:** 1 Cardiology, University of Florida College of Medicine, Jacksonville, USA; 2 Cardiology, Saint Louis University School of Medicine, Saint Louis, USA

**Keywords:** remodeling, echocardiography, aortic stenosis, tavi, tavr

## Abstract

Background

Transcatheter aortic valve replacement (TAVR) is increasingly utilized for most patients with symptomatic severe aortic stenosis. TAVR is linked to enhanced long-term cardiac hemodynamics, reversal of left ventricle (LV) hypertrophy, and improved aortic valve gradients. We present a retrospective observational study assessing cardiac remodeling and valvular flow patterns post-TAVR.

Methods

Retrospective echocardiographic data were collected, evaluating cardiac function and valvular flow patterns before and after TAVR at a single institution. Data was compiled and statistically analyzed using a paired t-test evaluating variations at approximately 30 days and one-year post-TAVR.

Results

On echocardiogram 30 days and one-year post-TAVR, there was a reduction in LV mass index from 132 g/m² to 110 g/m² (95%CI: 98-122; p=0.01) and 118 g/m² (95%CI: 102-133; p=0.03), and a reduction in relative wall thickness from 0.54 to 0.49 (95%CI: 0.46-0.52; p=0.05) and 0.44 (95%CI: 0.38-0.49; p=0.03), respectively. Doppler velocity indices (DVI) increased from 0.24 to 0.61 (95%CI: 0.49-0.73; p<0.001) and 0.57 (95%CI: 0.48-0.65; p<0.001). Expected improvement in aortic valve velocities and gradients were observed post-TAVR.

Conclusions

Following TAVR, LV remodeling can be observed as early as 30 days. This is demonstrated by a reduction in LV mass index and relative wall thickness in conjugation with an anticipated improvement in valvular flow patterns and flow across the aortic valve.

## Introduction

Since 2011, the PARTNER (Placement of AoRTic TraNscathetER Valve Trial) has paved the way demonstrating that transcatheter aortic valve replacement (TAVR) was equal in outcomes to surgical aortic valve replacement (SAVR) in severe aortic stenosis (AS) patients of high surgical risk [1]. In 2019, the benefits of TAVR have expanded to include patients of low surgical risk, demonstrating lower rates of death, stroke, or rehospitalization at one year compared to SAVR [2]. The 2020 AHA/ACC Valve guidelines went on to specifically recommend transfemoral TAVR in preference to SAVR in severe AS patients who are >80 years old in the presence of suitable vascular anatomy [3]. Over the next decade, it can be expected that TAVR frequency increase as treatment of choice for many severe AS patients, excluding patients with severe AS who are <65 years of age or have a life expectancy >20 years [3].

Various studies have gone on to examine long-term clinical outcomes [4,5] and valve durability and structural integrity [6] post-TAVR. Echocardiography has demonstrated that TAVR is associated with a reduction of the aortic valve gradients, improved hemodynamics, and regression of left ventricle (LV) hypertrophy [7,8]. Yet, data describing the prosthetics aortic valve’s hemodynamics and cardiac remodeling patterns over time is limited within the published literature. Echocardiography is the recommended imaging modality of choice in assessing valvular performance, cardiac function, and long-term follow-up [9]. Therefore, we aimed to perform a retrospective observational study examining cardiac remodeling and valvular flow patterns 30 days up to one year following TAVR at a single institution.

## Materials and methods

Study design

A retrospective observational study was performed evaluating electronic medical records and echocardiographic data of patients that had undergone TAVR at a single institution (Saint Louis University, Missouri, USA), a tertiary-care hospital and referral center serving urban, suburban, and rural communities. The Saint Louis University Institutional Review Board approved the protocol (protocol number: 30365). No patient consent was obtained as the study design qualified as secondary research involving only medical records review. All collected patient and echocardiographic data were de-identified.

Study outcome

The primary outcome of the study was to observe the reduction in LV mass index, in addition to the expected increase in the aortic valve area and decrease in aortic velocities and gradients post-TAVR. Secondary outcomes included the evaluation of all other cardiac remodeling and valvular flow patterns over time.

Patient selection

Between August 2015 and September 2019, 129 adult patients underwent TAVR at our institution. All patients’ electronic medical records and echocardiographic data were evaluated for inclusion in the study. Echocardiographic data needed to be available both before and after TAVR; data post-TAVR would need to be available at approximately 30-days, one-year, or at both time points. Patients with missing echocardiographic data were excluded. These included patients who were deceased post-TAVR or patients referred for TAVR that were being followed by physicians at outside institutions. Patients that had undergone any type of aortic valve implantation/replacement procedure (TAVR or SAVR) prior to the documented TAVR were excluded (11 patients). Following records evaluation, 66 patient records met the inclusion criteria for the study.

Data collection

At the institution, prior to undergoing TAVR, patients would require an echocardiogram for the evaluation of aortic stenosis. Once the TAVR was completed a post-TAVR echocardiogram was scheduled at approximately 30 days to re-evaluate the aortic valve’s hemodynamics and cardiac function. This would then be followed by long-term follow-up with the primary physician or Cardiologist, who may or may not re-order an echocardiogram at approximately one-year, based on clinical indication.

Data on patient characteristics at the time of TAVR were collected from the electronic medical records. This data included age, sex, social history, medical comorbidities, functional status, and the type of implanted aortic valve. Echocardiography data were extracted from the electronically available imaging and numeric data. Echocardiograms were performed by in-patient or out-patient echocardiogram laboratories. As the data were collected retrospectively, there was no standardization for sonographic acquisition or interpretation of echocardiograms.

Data were collected on Society of Thoracic Surgeons (STS) and Kansas City Cardiomyopathy Questionnaire (KCCQ) scores before TAVR. The STS score is used to calculate a patient's risk of mortality and morbidity from cardiac surgery [10]. The KCCQ score is a tool used to provide an understanding of a heart failure patient’s quality of life and predict clinical outcomes over time. Its domains include the patient’s physical limitations, symptoms description, self-efficacy, knowledge, social interference, and health-related quality of life. It is based on a 0 to 100 point scale: 0 to 24: very poor to poor; 25 to 49: poor to fair; 50 to 74: fair to good; and 75 to 100: good to excellent [11].

Statistical analysis

Data were evaluated for normal distribution using box and Q-Q plot analysis, and extreme outliers were rejected using the Inter Quartile Range method. Continuous echocardiographic numerical variables were statistically analyzed using a paired t-test evaluating variations at approximately 30 days and one-year post-TAVR. All testing used a two-sided alpha level of 0.05. Continuous variables are presented as mean ± standard deviation or along with 95% confidence intervals where applicable. All statistical analyses were performed with the use of R Studio version 1.2.1335, part of the R statistical software [12].

## Results

The majority of patients included in the study were older (mean 76 years old) and had hypertension (94%). The distribution of females to males was approximately equal (53% female, 47% male). Patients pre-TAVR had intermediate STS scores (between 4% and 8%) and poor to fair mean KCCQ scores (45, 95% confidence interval [CI]: 39-52) [Table 1].

**Table 1:** Patient Characteristics ACS: acute coronary syndrome, Afib or Aflut: atrial fibrillation or atrial flutter, CABG: coronary artery bypass grafting, CAD: coronary artery disease, CKD: chronic kidney disease, COPD: chronic obstructive pulmonary disease, CVA/TIA: cerebrovascular accident/transient ischemic attack, DM: diabetes mellitus, HLD: hyperlipidemia, HTN: hypertension, KCCQ: Kansas City Cardiomyopathy Questionnaire score, NYHA: New York Heart Association, OSA: obstructive sleep apnea, PM or ICD: pacemaker or implantable cardioverter-defibrillator, Pulm HTN: pulmonary hypertension, PVD: peripheral vascular disease, SD: standard deviation, STS: Society of Thoracic Surgeons score

(Total: n = 66)			
	Mean ± SD		n (%)
Age, years	76 ± 10	PM or ICD	8 (12)
STS score	5.8 ± 5.3	CAD	43 (65)
KCCQ: pre-TAVR	45 ± 27	ACS	18 (27)
KCCQ: 30-day post-TAVR	76 ± 24	Coronary stent	17 (26)
KCCQ: 1-year post-TAVR	73 ± 26	CABG	12 (18)
		CVA/TIA	11 (17)
	n (%)	CKD	19 (29)
Sex:		PVD	5 (8)
Male	31 (47)	COPD	20 (30)
Female	35 (53)	Pulm HTN	9 (14)
Smoking status:		OSA	20 (30)
Current	4 (6)	NYHA class:	
Former	37 (56)	I	1 (2)
Never	25 (38)	II	26 (39)
Alcohol use	21 (32)	III	34 (52)
HTN	62 (94)	IV	5 (8)
HLD	35 (53)	Valve used:	
DM	34 (52)	CoreValve	57 (86)
Afib or Aflut	18 (27)	Edwards-S3	9 (14)

Echocardiographic data were organized into timepoint following TAVR: first echocardiogram (mean: 33 days) and post-TAVR follow-up echocardiogram (mean: 327 days). On 30-day and one-year follow-up echocardiograms post-TAVR, there was a reduction in LV mass index from 132 g/m² to 110 g/m² (95%CI: 98-122; p=0.01) and 118 g/m² (95%CI: 102-133; p=0.03) and a reduction in relative wall thickness from 0.54 to 0.49 (95%CI: 0.46-0.52; p=0.05) and 0.44 (95%CI: 0.38-0.49; p=0.03), respectively. KCCQ scores improved from 45 to 76 on 30-day (95%CI: 69-83; p<0.001) and 73 on one-year (95%CI: 64-82; p<0.001) follow-up post-TAVR. 

A predictable reduction in aortic valve velocities and gradients was observed. In addition, to an expected post-prothesis implantation increase in doppler velocity indices (DVI) from 0.24 to 0.61 (95%CI: 0.49-0.73; p<0.001) and 0.57 (95%CI: 0.48-0.65; p<0.001) and LVOT (left ventricular outflow tract) peak velocity and gradient at 30-day and one-year follow-up. No statistically significant changes nor trends for improvement or deterioration were noted for aortic insufficiency, other valvular flow patterns, right ventricular function, or ejection fraction on follow-up echocardiograms [Table 2].

**Table 2:** Study Echocardiographic Findings *statistically significant, p-value <0.05 AI: aortic insufficiency, AV: aortic valve, CI: confidence interval, DVI: doppler velocity index, KCCQ: Kansas City Cardiomyopathy Questionnaire score, LA: left atrium, LV: left ventricle, LVOT: left ventricular outflow tract, MV: mitral valve, NA: not available, PASP: pulmonary artery systolic pressure, PV: pulmonary valve, RWT: relative wall thickness, SD: standard deviation, TAPSE: tricuspid annular plane systolic excursion, TAVR: transcatheter aortic valve replacement, VTI: velocity time integral

	Pre-TAVR echocardiogram (n=66)		Post-TAVR first echocardiogram (mean: 33 days) (n=62)			Post-TAVR follow-up echocardiogram (mean: 327 days) (n=31)		
	Mean ± SD	95% CI	Mean ± SD	95% CI	p-value	Mean ± SD	95% CI	p-value
LV mass, g	248 ± 110	(221, 276)	215 ± 73	(192, 234)	0.06	233	(197, 268)	0.07
LV mass index, g/m²	132 ± 56	(118, 146)	110 ± 39	(98, 122)	*0.01	118 ± 35	(102, 133)	*0.03
RWT	0.54 ± 0.14	(0.51, 0.58)	0.49 ± 0.10	(0.46, 0.52)	*0.05	0.44 ± 0.12	(0.38, 0.49)	*0.03
LV ejection fraction, %	58 ± 14	(54, 61)	57 ± 14	(53, 60)	0.76	56 ± 15	(50, 61)	0.34
AV mean gradient, mmHg	35 ± 14	(32, 38)	8 ± 4	(7, 9)	*<0.001	11 ± 5	(8, 13)	*<0.001
AV mean velocity, cm/s	276 ± 71	(259, 294)	127 ± 50	(95, 159)	*<0.001	154 ± 38	(134, 174)	*<0.001
AV peak gradient, mmHg	60 ± 24	(54, 65)	16 ± 8	(14, 18)	*<0.001	19 ± 8	(16, 22)	*<0.001
AV peak velocity, cm/s	376 ± 77	(358, 395)	187 ± 62	(171, 203)	*<0.001	215 ± 40	(200, 230)	*<0.001
AV VTI, cm	89 ± 26	(83, 96)	41 ± 11	(34, 47)	*<0.001	48 ± 12	(42, 54)	*<0.001
AV area, cm²	0.77 ± 0.21	(0.71, 0.82)	1.79 ± 0.47	(1.63, 1.95)	*<0.001	1.78 ± 0.47	(1.58, 1.97)	*<0.001
AV area index, cm²/m²	0.40 ± 0.12	(0.37, 0.43)	0.88 ± 0.22	(0.80, 0.96)	*<0.001	0.90 ± 0.23	(0.80, 1.00)	*<0.001
Aortic root diameter, cm	3.1 ± 0.5	(3.0, 3.3)	3.2 ± 0.5	(3.0, 3.3)	0.67	2.9 ± 0.6	(2.6, 3.1)	0.06
LVOT diameter, cm	2.1 ± 0.2	(2.0, 2.1)	2.1 ± 0.2	(2.0, 2.1)	0.56	2.0 ± 0.1	(2.0, 2.1)	0.09
LVOT peak velocity, cm/s	92 ± 27	(85, 98)	96 ± 27	(89, 104)	0.19	115 ± 31	(104, 127)	*<0.001
LVOT peak gradient, mmHg	3.6 ± 2.2	(3.1, 4.2)	4.9 ± 2.4	(3.3, 6.5)	0.088	6.0 ± 3.1	(4.5, 7.5)	*0.02
LVOT mean velocity, cm/s	71 ± 43	(61, 82)	79 ± 15	(69, 89)	0.10	63 ± 27	(50, 77)	0.68
LVOT mean gradient, mmHg	1.9 ± 1.0	(1.7, 2.2)	3.0 ± 1.2	(2.3, 3.8)	0.06	2.7 ± 1.2	(2.0, 3.3)	0.27
LVOT VTI, cm	20.7 ± 5.9	(19.2, 22.2)	22.3 ± 5.3	(19.2, 25.4)	0.57	24.3 ± 6.5	(21.4, 27.3)	0.48
LVOT stroke volume, ml	68 ± 22	(62, 73)	69 ±	(60, 79)	0.55	74 ± 28	(61, 88)	0.46
LVOT stroke volume index, ml/m²	35 ± 12	(32, 38)	35 ± 9	(30, 41)	0.61	39 ± 11	(34, 45)	0.96
LVOT cardiac output, ml/min	5110 ± 1570	(4720, 5500)	5364 ± 1550	(3440, 7290)	0.25	5560 ± 1270	(4390, 6740)	0.42
LVOT cardiac index, ml/min·m²	2680 ± 770	(2490, 2870)	2660 ± 900	(1540, 3770)	0.23	2750 ± 760	(2050, 3450)	0.19
DVI with LVOT VTI	0.24 ± 0.08	(0.22, 0.26)	0.61 ± 0.19	(0.49, 0.73)	*<0.001	0.57 ± 0.17	(0.48, 0.65)	*<0.001
AI peak velocity, cm/s	377 ± 51	(364, 390)	357 ± 20	(306, 407)	NA	365 ± 24	(335, 395)	0.095
AI peak gradient, mmHg	60 ± 16	(56, 63)	51 ± 6	(41, 60)	NA	54 ± 7	(45, 62)	0.11
AI pressure halftime, ms	344 ± 82	(325, 365)	448 ± 42	(344, 552)	NA	382 ± 137	(211, 552)	NA
MV peak velocity, cm/s	208 ± 159	(169, 248)	178 ± 55	(138, 217)	0.93	145 ± 35	(118, 172)	0.17
MV peak gradient, mmHg	9 ± 7	(8, 11)	14 ± 7	(9, 19)	0.58	11 ± 4	(8, 14)	0.49
Mitral E Point Velocity, cm/s	115 ± 42	(105, 126)	117 ± 45	(104, 129)	0.67	117 ±38	(102, 133)	0.86
Mitral A Point Velocity, cm/s	101 ± 49	(89, 113)	116 ± 43	(103, 129)	0.09	105 ± 38	(87, 123)	0.33
Mitral E to A Ratio	1.4 ± 0.9	(1.1, 1.6)	1.1 ± 0.6	(0.9, 1.2)	0.07	1.1 ± 0.7	(0.9, 1.4)	0.36
MV area, cm	3.1 ± 1.1	(2.9, 3.4)	2.3 ± 0.9	(1.5, 3.0)	0.57	2.8 ± 1.3	(1.9, 3.6)	0.07
PASP, mmHg	51 ± 16	(47, 55)	52 ± 14	(34, 69)	0.95	43 ± 7	(36, 50)	0.13
LA diameter, cm	4.5 ± 0.8	(4.3, 4.7)	4.3 ± 0.7	(4.1, 4.5)	0.11	4.6 ± 0.6	(4.4, 4.9)	0.31
LA volume, cm^3^	85 ± 37	(75, 94)	85 ± 38	(45, 125)	0.09	105 ± 21	(92, 119)	0.11
LA volume index, cm^3^/m^2^	44 ± 19	(40, 49)	41 ± 16	(26, 56)	0.17	55 ± 17	(46, 63)	0.08
PV Peak Velocity, cm/s	105 ± 27	(99, 112)	106 ± 28	(98, 114)	0.62	91 ± 24	(81, 101)	0.18
PV Peak Gradient, mmHg	4.7 ± 2.0	(4.2, 5.2)	4.1 ± 1.4	(3.3, 5.1)	0.69	3.4 ± 1.8	(2.5, 4.2)	0.18
TAPSE, mm	20 ± 4	(19, 21)	23 ± 3	(15, 30)	0.80	17 ± 4	(15, 20)	0.63
KCCQ	45 ± 27	(39, 52)	76 ± 24	(69, 83)	*<0.001	73 ± 26	(64, 82)	*<0.001

## Discussion

As anticipated, TAVR is associated with an immediate and sustained reduction in transaortic pressure gradients and an increase in the aortic valve area [13]. Flow-dependent parameters of AV peak velocity, peak gradient and mean gradient are expected to decrease over time, given changes in intravascular volume status, myocardial loading, and heart rate. While, flow-independent parameters aortic valve area (AVA), AVA index, and DVI are expected to not change on follow-up echocardiography compared to the first echocardiogram post-TAVR [7].

Aortic stenosis leads to LV pressure overload, LV hypertrophic remodeling, and an increase in relative wall thickness. Following TAVR, a hemodynamic improvement is expected following correction of stenosis and is complemented with immediate improvement in LV systolic function and gradual LV mass and relative wall thickness reduction [13]. In turn, this reveals LV plasticity and the ability for regression of LV hypertrophy over time. Prior studies have noted a regression in LV mass index after 30 days, one year [13], and five years [7]. Our data similarly demonstrated LV remodeling and regression in LV mass index on post-TAVR follow-up echocardiograms at 327 days. Early regression of LV mass index at 30 days post-TAVR has been associated with decreased hospitalization rates and improved morbidity at one year [14]. On 2-5 year follow-up, every 10% reduction in LV mass index is associated with a 5% reduction in all-cause mortality [15].

Despite a significant improvement in LV mass index, hemodynamic changes demonstrated a non-statistically significant trend towards improved LVOT stroke volume, stroke volume index, cardiac output, and cardiac index on follow-up echocardiograms, similar to prior studies [16]. Comparably, we did not observe an anticipated significant change in ejection fraction post-TAVR. Once deployed, it is expected for the TAVR to reduce myocardial contractility and impair diastolic function, followed by an improvement in ejection fraction after 30 days. This phenomenon is thought to be secondary to temporary myocardial stunning provoked by rapid ventricular pacing during TAVR [16].

Our finding of increased LVOT peak velocity and gradient on post-TAVR echocardiograms is likely secondary to the expected increase in peak velocity and gradient from the pre-stent LVOT location to the pre-leaflet in-stent location [7]. As evident by our results, ensuring accuracy is imperative in the measurements at pre-stent LVOT and pre-leaflet in-stent locations, as these measurements will determine the consistency of TAVR echocardiographic value performance evaluation. However, to better evaluate a TAVR prosthesis, the Doppler velocity index can be measured. DVI is a dimensionless ratio of the proximal LVOT velocity to the flow through the aortic valve prosthesis. It incorporates the effect of flow on velocity through the valve while being less dependent on valve size. It can be calculated as the ratio of the respective velocity-time integrals and approximated to the peak velocities. DVI is always less than one and a DVI of <0.25 is suggestive of significant prosthetists obstruction. Our patients had DVI 0.61 and 0.57 at 30-day and one-year echocardiograms, respectively. Thus, demonstrating lack of obstruction and possibly better outcomes as recent studies suggest that self-expanding valve DVI <0.50 following TAVR has higher three-year mortality (24.0% vs. 18.5%, p=0.025) [17].

Aortic insufficiency was not demonstrated on follow-up echocardiograms post-TAVR. Indeed, the absence of aortic insufficiency is supportive of good long-term valve durability, and its presence is associated with suboptimal late clinical outcomes and increased mortality [18]. In high and intermediate-risk patients, TAVR compared to surgical replacement has lower rates of 30-day mortality, stroke, and hospitalization, improved functional status, shorter duration of hospitalization, and lower rates of procedural complications [19]. Clinical outcomes on mortality and hospitalizations were not examined in our study; however, KCCQ scores on follow-up demonstrated significant improvement in heart failure patients’ quality of life, emphasizing TAVR’s meaningful impact on patient morbidity.

Study limitations

Our study was retrospective in design. Patients’ initial evaluation and echocardiograms pre- or post-TAVR were performed at our institution or other local institutions before referral to our institution. The available echocardiographic numerical and imaging data were limited. Echocardiograms were not performed in a single echocardiographic laboratory allowing for standardization, quality control of image analysis, and reproducibility, resulting in operator bias while performing and reading the echocardiograms. Moreover, transthoracic echocardiography may have prevented optimal evaluation of AV leaflet motion and possible subclinical valve thrombosis.

The sample size was notably decreased at one-year follow-up, possibly secondary to follow-up at other local institutions, loss to follow-up, or mortality. Data on mortality and hospitalizations was not available. The study was derived from data of a high-risk population with multiple comorbidities and a high mortality rate, which led to difficulty in long-term follow-up influenced by survival bias. Data was not available on hypertension management post-TAVR, as better blood pressure control may have played a role in LV remodeling over time. Future larger randomized prospective studies are needed to increase the quality of the echocardiographic and clinical data.

## Conclusions

TAVR demonstrates regression in LV mass index, relative wall thickness, and improvement in aortic valve flow patterns and hemodynamics. This leads to LV remodeling and regression of LV hypertrophy, which has been associated with decreased hospitalization rates, improved morbidity, and a reduction in all-cause mortality. Additionally, post-TAVR KCCQ scores demonstrated a clinically significant improvement in heart failure patients’ quality of life and morbidity. As TAVR continues to be the treatment of choice for the majority of severe aortic stenosis patients in the coming years, we will continue to further understand its associated echocardiographic remodeling patterns and long-term outcomes.
